# (−)-Asarinin from the Roots of *Asarum sieboldii* Induces Apoptotic Cell Death via Caspase Activation in Human Ovarian Cancer Cells

**DOI:** 10.3390/molecules23081849

**Published:** 2018-07-25

**Authors:** Miran Jeong, Hye Mi Kim, Jin Su Lee, Jung-Hye Choi, Dae Sik Jang

**Affiliations:** 1 College of Pharmacy, Kyung Hee University, Seoul 02447, Korea; jeongmiran@hanmail.net (M.J.); hyemi586@gmail.com (H.M.K.); lee2649318@naver.com (J.S.L.); 2 Department of Life & Nanopharmaceutical Sciences, Kyung Hee University, Seoul 02447, Korea

**Keywords:** *Asarum sieboldii*, (−)-asarinin, cytotoxicity, ovarian cancer cells, apoptosis, caspase

## Abstract

Two tetrahydrofurofurano lignans (**1** and **2**), four phenylpropanoids (**3**–**6**), and two alkamides (**7** and **8**) were isolated from the EtOAc-soluble fraction of the roots of *Asarum sieboldii*. The chemical structures of the isolates were identified by analysis of spectroscopic data measurements, and by a comparison of their data with published values. The isolates (**1**, **2**, **4**–**8**) were evaluated for their cytotoxicity against human ovarian cancer cells (A2780 and SKOV3) and immortalized ovarian surface epithelial cells (IOSE80PC) using a MTT (3-(4,5-dimethylthiazol-2-yl)-2,5-diphenyl-tetrazolium bromide) assay. Of the isolates, (−)-asarinin (**1**) exhibited the most potent cytotoxicity to both A2780 and SKOV3 cells. A propidium iodide/annexin V-fluorescein isothiocyanate (V-FITC) double staining assay showed that (−)-asarinin (**1**) induces apoptotic cell death in ovarian cancer cells. In addition, (−)-asarinin (**1**) increased the activation of caspase-3, caspase-8, and caspase-9 in ovarian cancer cells. Pretreatment with caspase inhibitors attenuated the cell death induced by (−)-asarinin (**1**). In conclusion, our findings show that (−)-asarinin (**1**) from the roots of *A. sieboldii* may induce caspase-dependent apoptotic cell death in human cancer cells.

## 1. Introduction

*Asarum sieboldii* Miq. (Aristolochiaceae) is a herbal plant that is widely distributed in Korea, China, and Japan. In traditional medicine, the roots of *A. sieboldii* are used as treatment for all types of colds, fever, chills, headaches, acute toothaches, sinusitis, cough, and dyspnoea, due to retention of phlegm, pharyngitis, chronic gastritis, and rheumatoid arthritis [1,2]. It has been demonstrated that *A. sieboldii* has a broad range of biological activity, such as anti-allergic [3], antitussive [4], anti-inflammatory [5], anti-nociceptive [6], anti-fungal [7,8], neuroprotective [9], and anticancer activity [10,11]. Previous phytochemical investigations on the roots of *A. sieboldii* resulted in the isolation of essential oils [12,13], alkamides [5,14], lignans [5,14], and alkaloids [15,16]. A 70% EtOH extract from *Asiasari radix* induced apoptosis preceded by a tight cell cycle arrest in the G2/M phase, suggesting that the extract prevented the growth of HCT-116 human colon cancer cells. The extract increased the expression of Bax/Bcl-2 and p53, and activated caspases, including Caspase-9 and Caspase-8 [10]. In addition, *Asiasari radix* extract significantly enhanced the sensitivity of HeLa cells to paclitaxel [17]. Moreover, Park et al. have demonstrated that the EtOAc-soluble fraction of *Asiasari radix* exhibits cytotoxic activity against human cancer cell lines such as A549 (human lung cancer), SKOV3 (human ovarian cancer), and SKMEL-2 (human melanoma) [11]. As part of an our ongoing project to search for novel, plant-derived anti-cancer agents [18], we found that the EtOAc-soluble fraction of the 70% EtOH extract of *A. sieboldii* roots exhibited significant cytotoxicity in the human ovarian cancer cells A2780 and SKOV3. Fractionation of the active EtOAc-soluble fraction resulted in the isolation and identification of eight known compounds consisting of tetrahydrofurofurano lignans (**1** and **2**), phenylpropanoids (**3**–**6**), and alkamides (**7** and **8**). The structures of the isolates were determined by spectroscopic analyses, including ^1^H-NMR, ^13^C-NMR, 2D-NMR, and MS spectra, and via a comparison of the data with published values. The isolates (**1**, **2**, **4**–**8**) were evaluated for their cytotoxicity against human ovarian cancer cells (A2780 and SKOV3) and immortalized ovarian surface epithelium cells (IOSE80PC), using MTT (3-(4,5-dimethylthiazol-2-yl)-2,5-diphenyl-tetrazolium bromide) assays. Of the isolates, a tetrahydrofurofurano lignin (−)-asarinin (**1**), which displayed potent cytotoxicity against both A2780 and SKOV3 cells, was investigated for the molecular mechanism of its cytotoxic activity. Here, we describe the isolation and identification of the isolates (**1**–**8**) from the roots of *A. sieboldii*, as well as their cytotoxicity to human ovarian cancer cells. This paper also deals with a mechanism study for cytotoxicity of (−)-asarinin (**1**) in human cancer cells.

## 2. Results

### 2.1. Cytotoxicity of the Extract and Solvent Fractions against Human Ovarian Cancer Cells

The 70% EtOH extract of the roots of *A. sieboldii* and two solvent partitions (EtOAc- and water-soluble fractions) from the 70% EtOH extract were investigated for their cytotoxicity against human ovarian cancer cells (A2780 and SKOV3) using MTT assays (Table 1). The 70% EtOH extract showed a significant cytotoxicity against A2780, with an observed IC_50_ value of 31.5 ± 16.83 μg/mL. Of the solvent partitions, the EtOAc-soluble fraction exhibited more potent cytotoxicity than the water-soluble fraction, against both ovarian cancer cells (IC_50_ values were 19.89 and 118.47 μg/mL in A2780 and SKOV3, respectively). Thus, we attempted to identify the cytotoxic constituents in the EtOAc-soluble fraction. Our data on the EtOAc-soluble fraction were consistent with previous results [11] on the cytotoxic activity of the EtOAc-soluble fraction of *Asiasari radix* against several human cancer cell lines including SKOV3 cells.

### 2.2. Identification of Compounds ***1***–***8*** from the Roots of A. sieboldii

**Figure 1:**
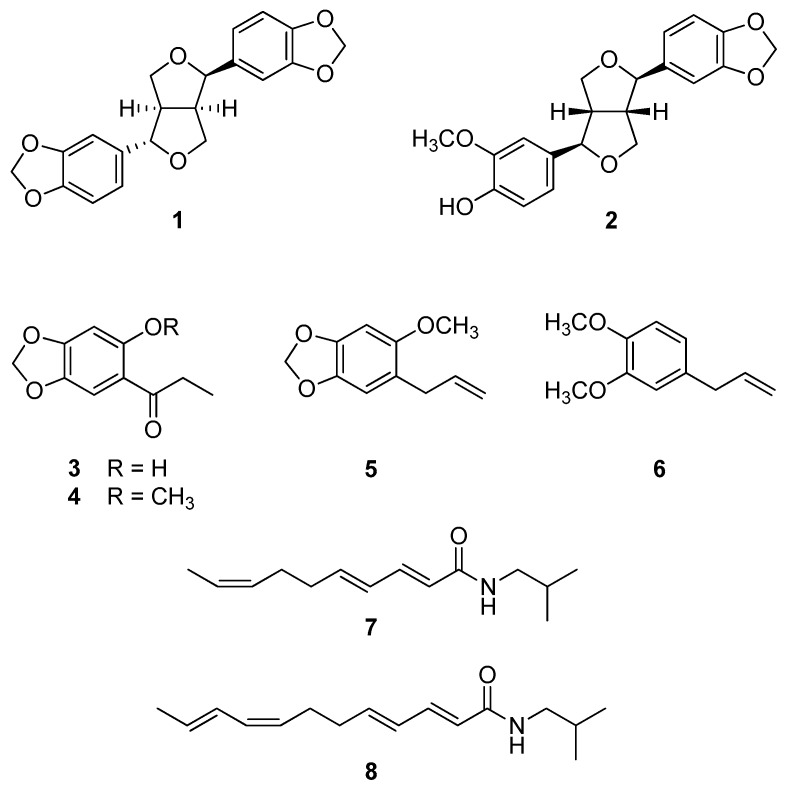
Compounds **1**–**8** isolated from the EtOAc-soluble fraction of the roots of *A. sielboldii*.

Two tetrahydrofurofurano lignans (**1** and **2**), four phenylpropanoids (**3**–**6**), and two alkamides (**7** and **8**) were isolated from the active EtOAc-soluble fraction of the roots of *A. sieboldii*. The chemical structures of the isolates were identified to be (−)-asarinin (**1**) [19], (−)-pluviatilol (**2**) [20], kakuol (**3**) [21], methylkakuol (**4**) [21], asaricin (**5**) [22], methyleugenol (**6**) [23], (2*E*,4*E*,8*Z*)-*N*-isobutyldeca-2,4,8-trienamide (**7**) [23], and (2*E*,4*E*,8*Z*,10*E*/*Z*)-*N*-isobutyldodeca-2,4,8,10-tetraenamide (**8**) [24], by analysis of spectroscopic data (^1^H-NMR, ^13^C-NMR, 2D-NMR, and MS) measurements, and by a comparison of their data with published values (Figure 1).

### 2.3. Cytotoxicity of Compounds ***1***–***8*** against Human Ovarian Cancer Cells

To identify compounds with cytotoxic activity against human cancer cells from the *A. sieboldii* roots, we investigated the effect of the isolates (**1**, **2**, **4**–**8**) obtained from the EtOAc-soluble fraction of the roots of *A. sieboldii* in human ovarian cancer cells A2780 and SKOV3. The effects of the isolates were assessed using IC_50_ values and they are summarized in Table 2. Of these, a tetrahydrofurofurano lignin, (−)-asarinin (**1**), exhibited the most potent cytotoxicity on both A2780 and SKOV3 cells, with observed IC_50_ values of 38.45 ± 2.78 and 60.87 ± 5.01 μM, respectively. Interestingly, (−)-asarinin (**1**) did not show any cytotoxicity against immortalized ovarian surface epithelial IOSE80PC cells, which were used as a normal counterpart of ovarian cancer cells. On the other hand, another lignan (−)-pluviatilol (**2**) showed a mild cytotoxicity against all the three cells tested (A2780 (IC_50_ value of 101.85 ± 13.55 μM), SKOV3 (IC_50_ value of 173.82 ± 9.42 μM), and IOSE80PC cells (IC_50_ value of 178.92 ± 3.30 μM)). An alkamide, (2*E*,4*E*,8*Z*,10*E*/*Z*)-*N*-isobutyldodeca-2,4,8,10-tetraenamide (**8**), exhibited a mild cytotoxicity only against A2780 cells (IC_50_ value of 101.20 ± 10.35 μM), but not against SKOV3 cells. These results implicate that (−)-asarinin (**1**), one of major constituents of the roots of *A. sieboldii*, seems to contribute to the cytotoxicity of the EtOAc-soluble fraction against cancer cells. (−)-Sesamin, which is an epimer of asarinin and is also found in this plant [5], exerts cytotoxic activity toward cancer cells and induces apoptosis in vitro [25,26,27,28]. An anticancer effect of (−)-sesamin in athymic mice transplanted with human MCF-7 breast cancer cells has also been reported [25]. Furthermore, the dietary supplementation of (−)-sesamin on 7,12-dimethylbenz[*a*]anthracene (DMBA)-induced breast carcinogenesis led to a significantly reduced appearance of breast cancers, compared to mice that were not supplemented with (−)-sesamin [26]. In contrast, (−)-asarinin (**1**) has been suggested to have a significant inhibitory effect on EBV-EA activation and preserved the high viability of the Raji cells [29,30]. In addition, (−)-asarinin (**1**) inhibited DNA synthesis in human leukemia HL-60 cells [31]. However the effect of (−)-asarinin (**1**) on human solid cancer cell growth and its underlying molecular mechanism have not been reported to date.

### 2.4. (−)-Asarinin (***1***)-Induced Apoptotic Cell Death in Human Ovarian Cancer Cells

**Figure 2:**
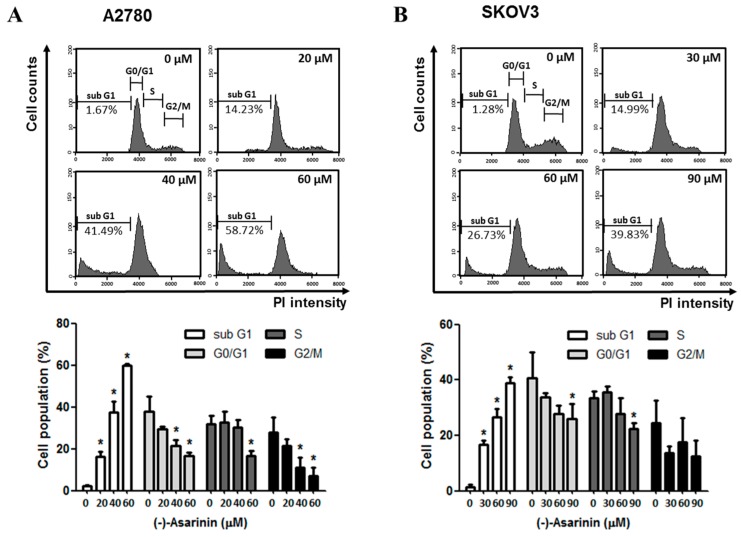

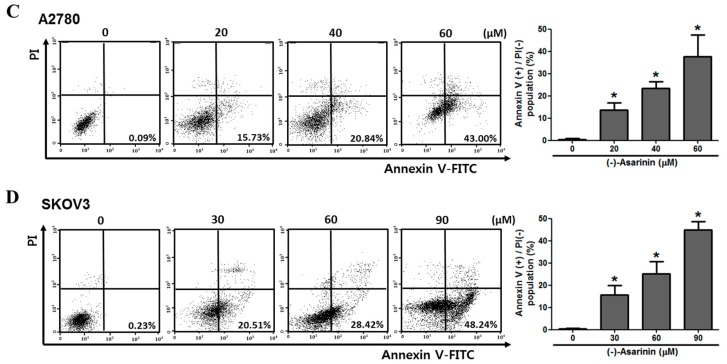
The effect of (−)-asarinin (**1**) on the cell cycle and apoptosis of human ovarian cancer cells. (**A** and **B**) A2780 cells (**A**) and SKOV3 cells (**B**) were treated with (−)-asarinin (**1**) for the indicated concentrations for 48 h. The cells were stained with propidium iodide (PI) according to the protocol as described in method. The cell cycle distribution profiles were measured by flow cytometry. FACS images shown are representative of three independent experiments. Means ± SD from three independent experiments are graphed from the proportion of cells (%) in each phase of cell cycle (sub G1, G0/G1, S, and G2/M). Statistical significance was determined by one-way ANOVA. * *p* < 0.05 as compared with the untreated group. (**C** and **D**) A2780 cells (**C**) and SKOV3 cells (**D**) were treated with the indicated concentration of (−)-asarinin (**1**) for 48 h. Apoptotic cell death analysis was performed using PI/Annexin V-fluorescein isothiocyanate (V-FITC) double staining assay. The data are representative of three independent experiments. Statistical significance was determined by a one-way ANOVA. * *p* < 0.05 as compared with the untreated group.

We found that (−)-asarinin (**1**) increased the fractionation of the nuclei accumulated at Sub G1 in A2780 and SKOV3 cells, but it failed to induce cell cycle arrest, which is one mechanism of growth inhibition (Figure 2A,B). Therefore, we further explored the molecular mechanism of the cytotoxic activity of (−)-asarinin (**1**) in human ovarian cancer cells. Dysfunction of apoptosis signaling pathways is associated with cancer development [32]. Therefore, compounds that promote apoptosis in cancer cells are considered as good candidates for anti-cancer chemotherapeutics. To examine whether (−)-asarinin (**1**)-induced cell death is mediated by the induction of apoptosis, two ovarian cancer cells A2780 and SKOV3 cells were treated with (−)-asarinin (**1**) and subsequently co-stained with PI and Annexin V-FITC. Treatment with (−)-asarinin (**1**) increased the percentage of Annexin V-FITC positive/PI negative cells by up to 43% in A2780 cells and 48% in SKOV3 cells (Figure 2C,D). These results suggest that the (−)-asarinin (**1**)-induced cell death is associated with the induction of apoptosis in human ovarian cancer cells.

### 2.5. (−)-Asarinin (***1***) Induced Caspase-Dependent Cell Death in Human Ovarian Cancer Cells

**Figure 3:**
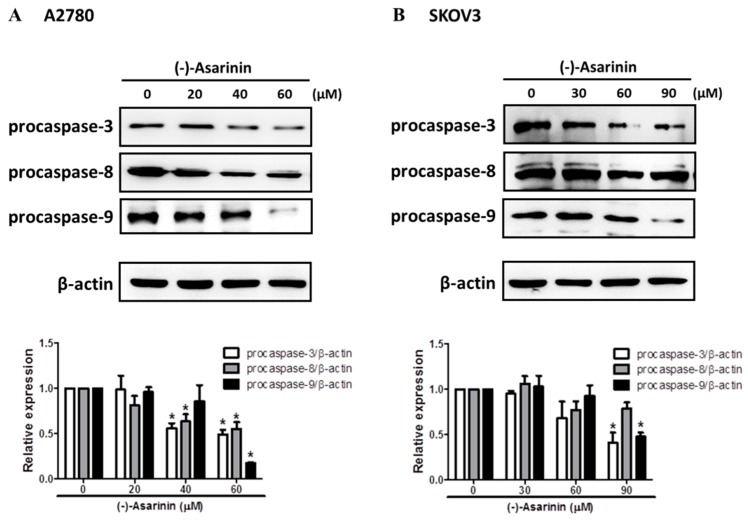
The effect of (−)-asarinin (**1**) on caspase activation in human ovarian cancer cells. A2780 (**A**) and SKOV3 (**B**) cells were treated with the indicated concentration of (−)-asarinin (**1**) for 48 h. Procaspase-3, -8, and -9 levels were determined by Western blot assay. β-Actin was used as an internal control. A representative protein immunoblot of three independent experiments is shown. Data are presented as the means ± SD of three independent experiments. Statistical significance was determined by one-way ANOVA. * *p* < 0.05 as compared with the untreated group.

**Figure 4:**
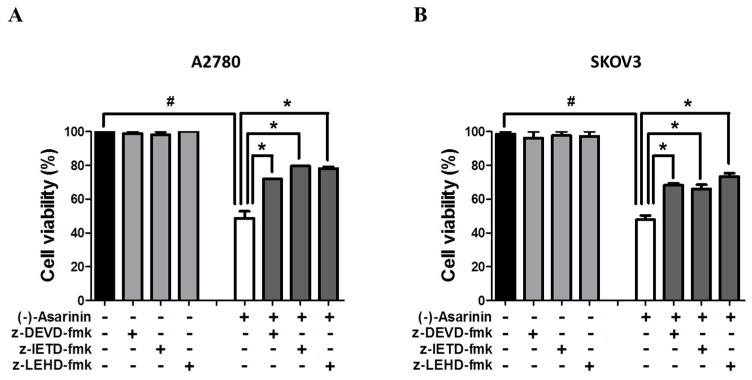
The effect of caspase inhibitors on (−)-asarinin (**1**)-induced cell death in human ovarian cancer cells. A2780 (**A**) and SKOV3 (**B**) cells were pretreated with caspase-3 inhibitor z-DEVD-fmk (50 μM), caspase-8 inhibitor z-IETD-fmk (50 μM), and caspase-9 inhibitor z-LEHD-fmk (50 μM) for 30 min, and then treated with the indicated concentration (A2780; 40 μM and SKOV3; 60 μM) of (−)-asarinin (**1**) for 48 h. A MTT (3-(4,5-dimethylthiazol-2-yl)-2,5-diphenyl-tetrazolium bromide) assay was performed to determine the cell viability after (−)-asarinin (**1**) treatment. Statistical significance was determined by Student’s *t*-test. # *p* < 0.05 as compared with the untreated group and * *p* < 0.05 as compared with the (−)-asarinin (**1**) only-treated group.

The activation mechanisms that trigger apoptosis are often referred to as the extrinsic and intrinsic pathways [33,34]. The intrinsic pathway (also called the mitochondria-mediated pathway) is activated directly by a variety of intracellular death stimuli. On the other hand, the extrinsic pathway (also called the receptor-mediated pathway) is characterized by the activations of cell-surface death receptors by the binding of extracellular ligands. Both extrinsic and intrinsic apoptotic pathways are highly dependent on the activation of caspases, which play a critical role in the proteolysis of specific targets [35]. Caspase-8 and caspase-9 are the initiator caspases for the extrinsic and intrinsic apoptotic pathways, respectively, while the effector caspase caspase-3 can be stimulated by activation of the initiator caspases in both pathways. To identify the mechanisms involved in (−)-asarinin (**1**)-induced apoptotic cell death, we investigated the activation of an effector caspase, caspase-3 and initiator caspases caspase-8 and caspase-9. (−)-Asarinin (**1**) markedly stimulated the activation of caspase-3, caspase-8, and caspase-9 in both A2780 and SKOV3 cells (Figure 3). To further confirm the involvement of caspases in (−)-asarinin (**1**)-induced apoptosis, the effect of caspase inhibitors on (−)-asarinin (**1**)-induced cell death was investigated. z-DEVD-fmk (a specific caspase-3 inhibitor), z-IETD-fmk (a specific caspase-8 inhibitor), and z-LEHD-fmk (a specific caspase-9 inhibitor) considerably attenuated (−)-asarinin (**1**)-induced cell death in both A2780 and SKOV3 cells (Figure 4). These results show that (−)-asarinin (**1**)-induced apoptosis is mediated by the caspase-dependent pathway in human ovarian cancer cells.

## 3. Materials and Methods

### 3.1. General Procedures

Open column chromatography (CC) was performed with silica gel (70–230 or 230–400 mesh ASTM, Merck, Kenilworth, NJ, USA), Sephadex LH-20 (Amersham Pharmacia Biotech, Piscataway, NJ, USA), reversed-phase silica gel (ODS-A 12 nm S-75 μm, YMC Co., Tokyo, Japan), and Redi Sep-C18 (26 g, Teledyne Isco, Lincoln, NE, USA). Thin-layer chromatography (TLC) was performed on Silica gel 60 F254 (Merck) and RP-18 F254S (Merck) plates; compounds were visualized by ultraviolet (UV) light (254 and 365 nm) and 20% (*v*/*v*) H_2_SO_4_ reagent (Aldrich, St. Louis, MI, USA). NMR spectra were recorded on a Bruker (Boston, MA, USA) 400 MHz and Varian 500 MHz NMR spectrometer using TMS as an internal standard, and chemical shifts were expressed as δ values. All solvents used for the chromatographic separations were distilled before use.

### 3.2. Plant Meterial

The roots of *Asarum sieboldii* Miq. (Aristolochiaceae) were purchased at Miryon Herbal Medicine Co. Gyeonggi-do, Korea, in March, 2013. Plant material was identified by Prof. Dae Sik Jang, one of the authors. A voucher specimen (No. 2013-ASSI01) has been deposited in the Lab of Natural Product Medicine, College of Pharmacy, Kyung Hee University, Republic of Korea.

### 3.3. Extraction and Isolation

The dried roots of *A. sieboldii* (280.4 g) was extracted with 70% EtOH (3.65 L) three times at room temperature, each for 6 h. The extract (40.15 g) was suspended in H_2_O (350 mL) and successively extracted with EtOAc (350 mL × 3) to yield EtOAc- (5.73 g) and a water-soluble fraction (34.92 g), respectively. The EtOAc-soluble fraction (5.73 g) was chromatographed over silica gel CC (70–230 mesh, ø 4.7 × 38.5 cm) as stationary phase with *n*-hexane-EtOAc-MeOH (9:1:0–0:0:1, *v*/*v*) as a mobile phase to generate 14 fractions (E1~E14). Fraction E3 (260.0 mg) was subjected to silica gel CC (230–400 mesh, ø 3.7 × 22.5 cm) with an *n*-hexane-CH_2_Cl_2_-EtOAc mixture (7:3:0.1, *v*/*v*) to give compound **5** (1.3 mg). Compounds **1** (344.4 mg), **2** (20.0 mg), and **3** (45.2 mg) were obtained by recrystallization (in *n*-hexane) from fractions E6 (274.1 mg), E9 (161.3 mg), and E4 (847.3 mg), respectively. Fraction E8 (740 mg) was further subjected to Sephadex LH-20 CC (ø 3.6 × 73.5 cm) eluted with CH_2_Cl_2_-MeOH mixture (1:1, *v*/*v*), to produce six subfractions (E8-1~E8-6). Compounds **7** (4.0 mg) and **8** (100.0 mg) were isolated from subfraction E8-2 (530 mg) by reversed-phase CC (YMC gel 75 μm, ø 3.6 × 23.5 cm) with MeOH-H_2_O (4:1, *v*/*v*). Fraction E4 (847 mg) was separated using Sephadex LH-20 CC (ø 3.6 × 40 cm) eluted with CHCl_3_-MeOH (1:1, *v*/*v*), to give five subfractions (E4-1~E4-5). Compound **6** (487.5 mg) was obtained from subfraction E4-2 by a silica gel CC (230-400 mesh, ø 3.7 × 22.5 cm). Fraction E5 (271 mg) was fractionated using Sephadex LH-20 CC (ø 3.6 × 73.5 cm) with CH_2_Cl_2_-MeOH (1:1, *v*/*v*), to generate five subfractions (E5-1~E5-5). Compound **4** (29.8 mg) was purified from subfraction E5-4 (70 mg) by a flash chromatographic system with a Redi Sep-C18 column (26 g, MeOH-H_2_O = 7:13 to 3:2, *v*/*v*).

### 3.4. Cell Culture

Human ovarian cancer cell lines (A2780 and SKOV3 cells) were obtained from the American Type Culture Collection (ATCC), and immortalized ovarian surface epithelial cell lines (IOSE80PC) were provided by Dr. N. Auersperg (University of British Columbia, Vancouver, British Columbia, Canada) and Dr. A. Godwin (Fox Chase Cancer Center, Philadelphia, PA, USA). Cells were cultured in the Roswell Park Memorial Institute (RPMI) 1640, supplemented with penicillin (100 U/mL), streptomycin sulfate (100 μg/mL), and 5% fetal bovine serum (FBS) (Life Technologies, Inc., Grand Island, NY, USA) in a humidified atmosphere of 5% CO_2_–95% air at 37 °C.

### 3.5. MTT Assay

A MTT assay was performed to evaluate the cell viability. The MTT was obtained from Molecular Probes Inc. (Eugene, OR, USA). Briefly, the cells (1.0 × 10^5^/well) were seeded in a 96-well plate and incubated for 24 h. The cells were treated with extracts (0.125–200 μg/mL) and compounds (0.125–200 μM) and incubated for 48 h. MTT solution was added into each well (final concentration; 0.5 mg/mL) and the plates were incubated for an additional 4 h. The medium was removed and 50 μL of dimethyl sulfoxide (DMSO) was added. The optical density was measured at 540 nm using a microplate spectrophotometer (SpectraMax; Molecular Devices, Sunnyvale, CA, USA). To investigate the involvement of caspases in asarinin (**1**)-induced cell death, a MTT assay was also performed using caspase inhibitors. The cells were pretreated with caspase inhibitors (50 μM) for 30 min, and treated with (−)-asarinin (**1**) (A2780, 40 μM; and SKOV3, 60 μM) for 48 h. Caspase-3 inhibitor z-DEVD-fmk, caspase-8 inhibitor z-IETD-fmk, and caspase-9 inhibitor z-LEHD-fmk were purchased from Calbiochem (Bad Soden, Germany).

### 3.6. Cell Cycle Analysis

The cells were treated with (−)-asarinin (**1**) and incubated for 48 h. At the time of collection, the cells were harvested and washed twice with ice-cold phosphate buffered saline (PBS). The cells were fixed and permeabilized with 70% ice-cold ethanol at −20 °C for 4 h. The cells were washed once with PBS and resuspended in a staining solution containing propidium iodide (50 µg/mL) and RNase A (5 mg/mL). The cell suspensions were incubated for 30 min at room temperature in a dark place. After 30 min the suspensions were analyzed by fluorescence-activated cell sorting (FACS) cater-plus flow cytometry (guava easy cyte^TM^, Merk Millipore, Darmstadt, Germany) using 5000 cells per group.

### 3.7. Annexin V-FITC/PI Double Staining

Annexin V- FITC was obtained from BD Biosciences (San Jose, CA, USA). The cells were treated with (−)-asarinin (**1**) and incubated for 48 h. The cells were rinsed twice with ice-cold PBS and suspended with 100 μL of binding buffer (10 mM HEPES/NaOH, 140 mM Nacl, 2.5 mM CaCl_2_, PH 7.4). A total of 5 μL of FITC-conjugated annexin V and 5 μL of PI (50 mg/mL) were added into the cell suspension, and the mixture was incubated in a dark place at room temperature for 15 min. The cells were analyzed using FACS cater-plus flow cytometry (guava easy cyte^TM^); at least 10,000 cells per each group were counted.

### 3.8. Western Blot Assay

The cells were treated with (−)-asarinin (**1**) and incubated for 48 h. The cells were rinsed twice with ice-cold PBS and lysed with protein lysis buffer (Intron Biotechnology, Seoul, Korea) containing protease inhibitors (0.5 mM PMSF and 5 μg/mL aprotinin). The lysates were mixed with 5X sodium dodecyl sulfate (SDS) sample buffer and boiled for 5 min for denaturation. Total protein was run on 10–12% SDS-PAGE gels and electrotransferred onto a polyvinylidene difluoride (PVDF) membrane. The membrane was immunoblotted using specific primary antibodies overnight at 4 °C following blocking with 5% non-fat dry milk for 30 min–1 h. After washing, the membrane was incubated with horseradish peroxidase-conjugated secondary antibody (1:1000–2000) at room temperature for 1−2 h. After washing, immunepositive bands were visualized using an ECL chemiluminescent system and analyzed by Image Quant LAS-4000 (Fujifilm Life science, Tokyo, Japan). Anti-caspase-3 and b-actin antibodies were obtained from Santa Cruz Biotechnology (Santa Cruz, CA, USA). Caspase-9 antibody was purchased from Cell Signaling (Beverly, MA, USA). Caspase-8 antibody was obtained from BD Biosciences (San Jose, CA, USA).

### 3.9. Statistical Analysis

One-way ANOVA and Student’s *t*-test were performed to determine statistically significant differences. *p*-values of less than 0.05 were regarded as statistically significant.

## 4. Conclusions

Fractionation of the active EtOAc-soluble fraction from the 70% EtOH extract of the roots of *A. sieboldii* resulted in the isolation and identification of two tetrahydrofurofurano lignans (**1** and **2**), four phenylpropanoids (**3**–**6**), and two alkamides (**7** and **8**). Of the isolates, (−)-asarinin (**1**) exhibited the most potent cytotoxicity against two human ovarian cancer cells, A2780 and SKOV3, while it did not affect cell viability of normal human ovarian epithelial cells IOSE80PC. Treatment with (−)-asarinin (**1**) significantly induced apoptotic cell death in both A2780 and SKOV3 cells. We further demonstrated that (−)-asarinin (**1**) stimulated the activation of caspase-3, caspase-8, and caspase-9, and caspase inhibitors significantly reversed (−)-asarinin (**1**)-induced cell death in human ovarian cancer cells. Taken together, these results suggest that (−)-asarinin (**1**), a major component of the roots of *A. sieboldii*, induces apoptotic cell death specifically against cancer cells via the caspase-dependent pathway.

## Figures and Tables

**Table 1:** Cytotoxicity of the 70% EtOH extract of *A. sieboldii* roots and its solvent fractions in human ovarian cancer cells A2780 and SKOV3.

Extract or Fractions	IC_50_ (μg/mL) *	
	Human Ovarian Cancer Cells	
	A2780	SKOV3
70% EtOH extract	31.5 ± 16.83	>200
EtOAc fraction	19.89 ± 4.20	118.47 ± 19.78
Water fraction	107.20 ± 15.80	139.30 ± 29.13

* The IC_50_ value is defined as being a concentration that lowers cell viability by 50%. The value shows an average of the results of three independent experiments having similar patterns.

**Table 2:** Cytotoxicity of compounds **1**–**8** from the EtOAc-soluble fraction of the roots of *A. sieboldii* in human ovarian cancer cells (A2780 and SKOV3) and immortalized ovarian surface epithelial cells (IOSE80PC).

Compound	IC_50_ (μM) *		
	Human Ovarian Cancer Cells		Immortalized Ovarian Surface Epithelial Cells
	A2780	SKOV3	IOSE80PC
1	38.45 ± 2.78	60.87 ± 5.01	>200
2	101.85 ± 13.55	173.82 ± 9.42	178.92 ± 3.30
3	>200	>200	>200
4	>200	>200	>200
5	ND **	ND **	ND **
6	>200	>200	>200
7	>200	>200	>200
8	101.20 ± 10.35	>200	>200
Cisplatin ***	8.77 ± 0.48	24.18 ± 0.28	45.62 ± 0.30

* IC_50_ value is defined as the concentration that results in a 50% decrease in the proliferation of the cells. The values represent the means of the results from three independent experiments with similar patterns. ** Not determined since the amount of available compound was insufficient. *** Cisplatin was used as an assay positive control.
